# Development and validation of a nomogram for predicting ongoing pregnancy in single vitrified-warmed blastocyst embryo transfer cycles

**DOI:** 10.3389/fendo.2023.1257764

**Published:** 2023-11-23

**Authors:** Jae Kyun Park, Ji Eun Park, Soyoung Bang, Haeng Jun Jeon, Ji Won Kim, Woo Sik Lee

**Affiliations:** Department of Obstetrics and Gynecology, Fertility Center of CHA Gangnam Medical Center, CHA University School of Medicine, Seoul, Republic of Korea

**Keywords:** nomogram, prediction model, ongoing pregnancy outcome, single vitrified-warmed blastocyst embryo transfer, *in vitro* fertilization

## Abstract

**Introduction:**

The global adoption of the “freeze-all strategy” has led to a continuous increase in utilization of single vitrified-warmed blastocyst embryo transfer (SVBT) owing to its clinical effectiveness. Accurate prediction of clinical pregnancy is crucial from a patient-centered perspective. However, this remains challenging, with inherent limitations due to the absence of precise and user-friendly prediction tools. Thus, this study primarily aimed to develop and assess a nomogram based on quantitative clinical data to optimize the efficacy of personalized prognosis assessment.

**Materials and methods:**

We conducted a retrospective cohort analysis of ongoing pregnancy data from 658 patients with infertility who underwent SVBT at our center between October 17, 2017, and December 18, 2021. Patients were randomly assigned to the training (n=461) or validation (n=197) cohort for nomogram development and testing, respectively. A nomogram was constructed using the results of the multivariable logistic regression (MLR), which included clinical covariates that were assessed for their association with ongoing pregnancy.

**Results:**

The MLR identified eight significant variables that independently predicted ongoing pregnancy outcomes in the study population. These predictors encompassed maternal physiology, including maternal age at oocyte retrieval and serum anti-Müllerian hormone levels; uterine factors, such as adenomyosis; and various embryo assessment parameters, including the number of fertilized embryos, blastocyst morphology, blastulation day, blastocyst re-expansion speed, and presence of embryo string. The area under the receiver operating characteristic curve in our prediction model was 0.675 (95% confidence interval [CI], 0.622–0.729) and 0.656 (95% CI, 0.573–0.739) in the training and validation cohorts, respectively, indicating good discrimination performance in both cohorts.

**Conclusions:**

Our individualized nomogram is a practical and user-friendly tool that can provide accurate and useful SVBT information for patients and clinicians. By offering this model to patients, clinical stakeholders can alleviate uncertainty and confusion about fertility treatment options and enhance patients’ confidence in making informed decisions.

## 1 Introduction

Vitrification has enhanced the efficiency and safety of embryo cryopreservation considerably, allowing for extended prolonged storage of embryos and improving the overall success rate of assisted reproductive technology (ART) procedures, including *in vitro* fertilization (IVF) (1). Furthermore, extended culture, which involves *in vitro* embryo development up to the blastocyst stage before uterine transfer, enables the selection of competent embryos for transfer, enhancing the prospects of achieving a successful pregnancy (2). Advancements in vitrification and extended culture are crucial in increasing the success rate of ART, particularly blastocyst transfer (3, 4). Frozen embryo transfer (FET) has advanced notably in recent years; however, conception can remain challenging for individuals experiencing infertility. In addition to medical considerations, such as success rates, patients may face financial burdens associated with ART procedures (5). These factors, coupled with the emotional stress inherent in the process, can contribute to the overall psychological burden of attempting conception and potentially strain relationships (6).

Shared decision-making is a collaborative process between healthcare professionals and patients to make informed decisions concerning healthcare treatments and interventions. During this process, healthcare professionals present patients with comprehensive information on available treatment options, including the associated risks, benefits, and probable outcomes. Patients are encouraged to ask questions, articulate their preferences and concerns, and provide input for the decision-making process. Ultimately, healthcare professionals and patients decide the most appropriate treatment option tailored to each patient’s circumstances (7, 8). Implementing a nomogram model can help healthcare professionals better inform couples with infertility about their chances of conceiving (9, 10). A nomogram is a visual tool that simplifies the creation of a prediction model for disease diagnosis, recurrence, or survival by considering various risk factors without requiring complicated calculations. Nomograms are useful for predicting outcomes in various medical scenarios (11–14).

Owing to the specificity of IVF treatment field, the proportion of patients participating in decision-making is higher than that in other medical fields; therefore, it is patient-centered. Effective communication of treatment plans, including the risks, benefits, and potential complications, is critical before initiating FET treatment (8, 15). However, current treatment strategies often lack concrete scientific evidence, making counseling challenging and frustrating for couples with infertility seeking individualized care (16). To address this, our study aimed to optimize treatment success by estimating personalized probabilities using a nomogram of embryonic (blastocyst assessment) predictors and patient physiology (maternal physiology and uterine factor) before treatment.

## 2 Materials and methods

### 2.1 Ethical approval

This retrospective study was approved by the Ethics Committee of the Institutional Review Board (IRB) of the CHA Gangnam Medical Center (IRB approval number: GCI 2022-06-008). The need for obtaining participant consent was waived due to the study’s retrospective nature and the use of medical records. The entire study was conducted in accordance with relevant guidelines and regulations.

### 2.2 Study design and patients

**Figure 1:**
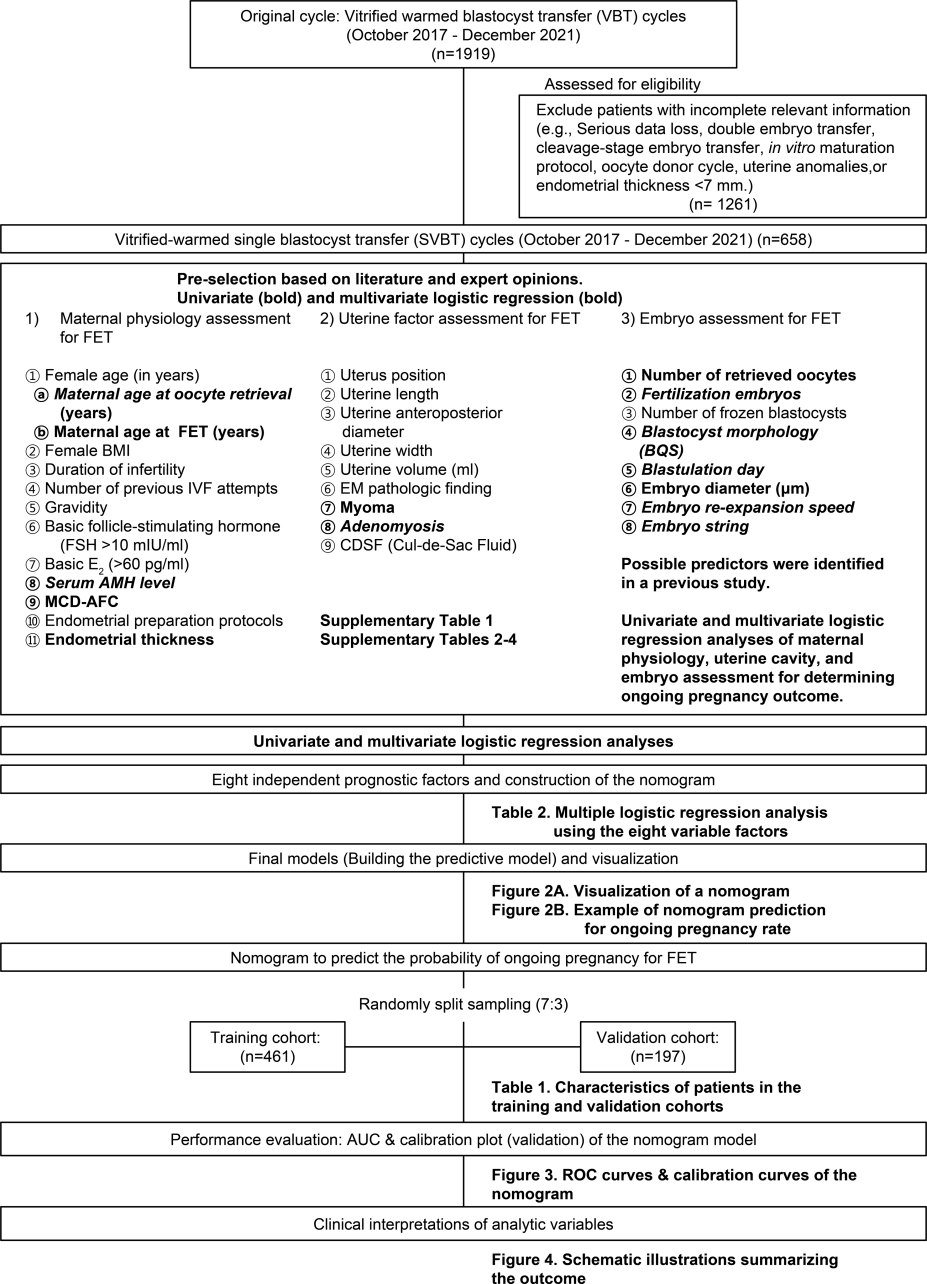
Flowchart of the experiment for predicting ongoing pregnancy after vitrified-warmed single embryo transfer cycles.

This retrospective cohort study was conducted at the CHA Fertility Center in Gangnam, South Korea, between October 17, 2017 and December 18, 2021. Overall, 1,919 single vitrified-warmed blastocyst embryo transfer (SVBT) cases were identified and analyzed. Demographic data and potential predictors were pre-selected based on a literature review and clinical experience (**Supplementary Table 1**). A detailed chart review confirmed embryo-related information. Exclusion criteria were double embryo transfer, cleavage-stage embryo transfer, *in vitro* maturation, oocyte donor cycle, uterine anomalies, endometrial thickness < 7 mm, not using the time lapse system (TLS), and significant data loss. The study flowchart (**Figure 1**) illustrates the selection process, resulting in the analysis of 658 SVBT cycles.

### 2.3 IVF and laboratory procedures

IVF and laboratory procedures were performed as previously described (17). The physician determined the dosage and duration of follicle-stimulating hormone (FSH) treatment based on patient information. Superovulation was induced using the gonadotropin-releasing hormone (GnRH) agonist long or antagonist protocol. The FSH dose was adjusted based on the growth of each follicle and serum estradiol (E_2_) concentration. Oocyte was retrieved under transvaginal ultrasound guidance when two or more follicles with a diameter of 17–18 mm or greater were identified following recombinant human chorionic gonadotropin administration. The retrieved oocytes were fertilized using conventional fertilization or intracytoplasmic sperm injection. A good-quality embryo was selected for transfer into the uterine cavity using a delivery catheter under abdominal ultrasound guidance. Surplus embryos, if available, were cryopreserved based on established protocols, considering criteria such as elevated progesterone or E_2_ levels or the risk of ovarian hyperstimulation syndrome. The vitrification and warming processes followed standardized methods as previously described (18, 19). Post-warming blastocyst survival was confirmed by assessing the morphological integrity using an inverted microscope (Vitrolife). Warmed blastocysts were cultured in TLS overnight, and their survival, re-expansion, and grading were evaluated based on the blastocyst quality score. Endometrial preparation for SVBT was conducted in natural or hormone replacement treatment cycles, as described in our previous study (20). In natural cycles, dominant ovarian follicle development was monitored, and SVBT was performed after ovulation. One day before embryo transfer. Vitrified blastocysts were warmed at 37°C. Upon confirmation of their survival and development, they were transferred to the uterine cavity under ultrasound guidance.

### 2.4 Development and validation of the model

Patient information was blinded, and they were assigned randomized numbers; a training (n=461) and a validation cohort (n=197) was established at a ratio of 7:3. Ongoing pregnancy, the endpoint of this study, was defined as the presence of a gestational sac with a heartbeat lasting for over 12 weeks. In the training cohort, 461 patients were included to develop a nomogram to predict the patient-specific probability of an ongoing pregnancy in women with SVBT. Backward stepwise conditional variable selection was performed to determine the independent covariates. A logistic regression model was used for multivariable analysis, including significant variables from the univariate analysis (*p < 0.05*). The variables entered into the nomogram development model were maternal age at oocyte retrieval, serum anti-Müllerian hormone (AMH) level, adenomyosis, number of fertilized embryos, blastocyst morphology score (BQS), vitrification day, blastocyst re-expansion speed (μm/min), and presence of an embryo string.

The values for each model covariate were mapped to points on a scale of 0–100, and the total points obtained for each model corresponded to the probability of an ongoing pregnancy. The performance of the model was assessed in the training cohort and internally validated by fitting it to the validation cohort using the same parameters. The predictive accuracy of the models was measured using the receiver operating characteristic (ROC) curve and the area under the curve (AUC). A calibration plot was used to plot the observed rates against the predicted probabilities to assess the agreement.

### 2.5 Statistical analysis

All statistical analyses were performed using IBM SPSS version 25.0 (IBM Corp., Armonk, NY, USA), R software (version 3.3.4, R Core Team, Vienna, Austria), and JAMOVI software (version 2.3, JAMOVI Project, Sydney, Australia). A *p-*value of < 0.05 was considered significant. Mean and standard deviation (SD) were used for normally distributed variables, chi-square for categorical variables, and t-test for continuous variables. First, univariate logistic regression analysis was performed on clinically important variables, and significant variables (*p* < 0.05) were potential predictors for the MLR. Step-by-step backward logistic regression analysis was conducted to obtain the final set of possible predictors. Two approaches were employed to assess the validity of the nomogram. First, discrimination or the ability of the nomogram to distinguish between ongoing pregnancy and non-pregnancy outcomes was quantified using the area under the receiver operating characteristic (AUROC) curve. The ROC curve provides a comprehensive evaluation of the nomogram’s predictive accuracy.

Second, calibration was assessed using a calibration plot. This plot compared the predicted probabilities from the nomogram with the observed probabilities of ongoing pregnancy, using the mean values of each group. A closer calibration plot line to the 45° angle corresponded with more accurate predictions. By evaluating discrimination and calibration, the utility and accuracy of the nomogram in predicting ongoing pregnancy could be determined.

Based on the initial analysis and previous studies, possible predictors were selected for the pregnancy prediction model. The characteristics were classified into three distinct phases: Phase 1, encompassing maternal physiology; Phase 2, centered on uterine factors; and Phase 3, revolving around embryo assessment. Elaborate details regarding these phases are in the **Supplementary Materials and Methods**.

## 3 Results

### 3.1 Description of the patient’s characteristics

The baseline patient characteristics are summarized in **Table 1**. In total, 658 patients were analyzed, with 461 and 197 in the training and validation cohorts, respectively. The two cohorts had no significant differences in the demographic characteristics or previous IVF cycles. The mean maternal age at FET was 34.1 ± 3.57 years. No significant differences were observed in endometrial thickness (9.61 ± 1.66 vs. 9.53 ± 1.80 mm) or preparation method between the training and validation cohorts. Furthermore, no significant difference was observed in both cohorts for infertility etiology. AMH (4.38 ± 3.65 vs. 3.98 ± 3.00 ng/mL), basal FSH (7.53 ± 3.01 vs. 7.30 ± 2.44 mlU/mL), and E_2_ levels (44.0 ± 20.4 vs. 45.3 ± 18.7 ρg/mL) also did not differ significantly in previous IVF cycles between both cohorts. Moreover, no significant differences existed in the number of retrieved oocytes (16.6 ± 8.88 vs. 16.7 ± 9.43), the number of fertilized embryos (11.1 ± 6.06 vs. 11.0 ± 6.2), or the number of frozen blastocysts (3.31 ± 2.48 vs. 3.33 ± 2.19) between the two cohorts.

**Table 1:** Patient characteristics in the training and validation cohorts.

	Variables	All	Training cohort	Validation cohort	*p*-value
Demographic characteristics	Number of cycles, n	658	461	197	
	Maternal age at FET (range)	34.1 ± 3.57 (21–47)	34.1 ± 3.67 (21–47)	34.3 ± 3.34 (25–46)	*0.813*
	Paternal age at FET (range)	36.6 ± 4.53 (25–68)	36.7 ± 4.58 (25–68)	36.3 ± 4.44 (27–62)	*0.890*
	Endometrial thickness (mm) (range)	9.59 ± 1.70 (5–15)	9.61 ± 1.66 (5–15)	9.53 ± 1.80 (5–15)	*0.851*
	Endometrial preparation, n (%)				
	Natural	537 (81.6%)	377 (81.8%)	160 (81.2%)	*0.986*
	HRT	121 (18.4%)	84 (18.2%)	37 (18.8%)	*0.986*
	Etiology of infertility, n (%)				
	Female	269 (40.9%)	187 (40.6%)	82 (41.6%)	*0.968*
	Male	125 (19.0%)	96 (20.8%)	29 (14.7%)	*0.188*
	Combined (male + female)	168 (25.5%)	111 (24.1%)	57(28.9%)	*0.426*
	Unexplained	80 (12.2%)	58 (12.6%)	22(11.2%)	*0.879*
	Other	16 (2.4%)	9 (2.0%)	7 (3.6%)	*0.475*
Characteristics of the previous IVF cycle	Maternal age at oocyte retrieval (range)	33.5 ± 3.61 (21–46)	33.5 ± 3.64 (21–45)	33.6 ± 3.55 (25–46)	*0.992*
	BMI (kg/m^2^) (range)	21.1 ± 2.62 (15.7–37)	21.2 ± 2.6 (15.7–35.8)	21.1 ± 2.76 (16–37)	*0.934*
	Duration of infertility (range)	3.19 ± 2.2 (1–15)	3.22 ± 2.17 (1–15)	3.11 ± 2.28 (1–12)	*0.856*
	Number of previous IVF attempts (range)	1.57 ± 1.23 (1–15)	1.56 ± 1.11 (1–12)	1.61 ± 1.46 (1–15)	*0.872*
	Gravidity, n (%)				
	0	605 (91.9%)	422 (91.5%)	183 (92.9%)	*0.843*
	≥1	53 (8.1%)	39 (8.5%)	14 (7.1%)	*0.843*
	MCD-AFC, n (range)	17.4 ± 10.3 (1–70)	17.6 ± 10.1 (1–70)	16.9 ± 10.8 (1–70)	*0.773*
	AMH (ng/mL) (range)	4.26 ± 3.47 (0.01–25.8)	4.38 ± 3.65 (0.14–25.8)	3.98 ± 3.00 (0.01–17.2)	*0.404*
	Basal FSH (mIU/mL) (range)	7.47 ± 2.86 (1.06–34.3)	7.53 ± 3.01 (1.06–34.3)	7.30 ± 2.44 (1.36–25.4)	*0.538*
	Basal E_2_ (ρg/mL) (range)	44.4 ± 19.9 (5–151)	44.0 ± 20.4 (5–151)	45.3 ± 18.7 (5–129)	*0.894*
	Mean number of retrieved oocytes (*n*) (range)	16.6 ± 9.04 (1–56)	16.6 ± 8.88 (1–56)	16.7 ± 9.43 (1–44)	*0.992*
	Mean number of fertilized embryos *(2 pronuclei)* (range)	11.0 ± 6.1 (1–40)	11.1 ± 6.06 (1–40)	11.0 ± 6.2 (1–33)	*0.995*
	Mean number of frozen blastocysts (n) (range)	3.32 ± 2.4 (1–16)	3.31 ± 2.48 (1–16)	3.33 ± 2.19 (1–12)	*0.997*

Values are presented as the mean ± standard deviation (SD). FET, frozen embryo transfer; HRT, hormone replacement treatment; BMI, body mass index; MCD, menstrual cycle day; AFC, antral follicle count; AMH, anti-Müllerian hormone; FSH, follicle-stimulating hormone; E_2_, estradiol hormone.

### 3.2 Association between eight predictor variables and ongoing pregnancy

First, independent variables associated with sustained pregnancy rates were evaluated in terms of maternal physiology, uterine factor, and embryo assessment. Regarding maternal physiology, univariate and multivariable logistic regression analyses revealed a significant association with maternal age at oocyte retrieval and serum AMH levels (**Supplementary Table 2**). However, only maternal age at FET, menstrual cycle day-antral follicle count (AFC), and endometrial thickness significantly correlated in the univariate logistic regression analysis. Female body mass index, duration of infertility, number of previous IVF attempts, gravidity, basal FSH level, basal E_2_ level, and endometrial preparation protocols were not associated with sustained pregnancy rates.

Similarly, the relationship between uterine factors and ongoing pregnancy was evaluated using univariate and multivariable logistic regression analyses. Adenomyosis was the only variable significantly associated with ongoing pregnancy (**Supplementary Table 3**). However, myoma only significantly correlated in the univariate logistic regression analysis. Uterine position, length, anteroposterior diameter, width, volume, endometrial (EM) pathology findings, and cul-de-sac fluid were not associated with sustained pregnancy rates.

Finally, we evaluated the relationship from the perspective of embryo assessment using univariate and multivariable logistic regression analyses. Of the 10 independent variables, the number of fertilized embryos, BQS, day of vitrification, re-expansion speed, and presence of embryo string were significantly correlated. The number of retrieved oocytes and embryo diameter were significant only in the univariate logistic regression analysis. The remaining variables, including fertilization method, number of vitrified blastocysts, and total vitrification, were not associated with sustained pregnancy rates (**Supplementary Table 4**).

The MLR results for the eight potential predictors of ongoing pregnancies are presented in **Table 2**. Eight predictors were significant: 1) maternal physiology (maternal age at oocyte retrieval and serum AMH level), 2) uterine factor (adenomyosis), and 3) embryo assessment (number of fertilized embryos, blastocyst morphology, blastulation day, blastocyst re-expansion speed, and presence of an embryo string).

**Table 2:** Multiple logistic regression analysis using eight variables (considering interactions).

Variables	Ongoing pregnancy OR (95% CI)	*p-value*	Adjusted OR (95% CI)	*p*-value
Maternal age at oocyte retrieval (years)				
≤37	2.439 (1.516–3.924)	*0.001*	2.331 (1.362–2.620)	*0.002*
≥38	Reference		Reference	
Serum anti-Mullerian hormone (AMH) level				
≤1.99	Reference		Reference	
≥2.0~≤4.0	1.568 (1.034–2.376)	*0.034*	1.673 (1.069–2.620)	*0.024*
≥4.1	1.941 (1.289–2.923)	*0.001*	1.917 (1.148–2.875)	*0.011*
Adenomyosis				
Yes	Reference		Reference	
No	3.801 (2.378–6.078)	*0.001*	3.801 (2.378–6.078)*	*0.001*
Number of fertilized embryos (*n*)				
≤7	Reference		Reference	
≥8–≤10	1.617 (1.049–2.492)	*0.029*	n/a	
≥11–≤14	1.011 (0.645–1.586)	0.962	n/a	
≥15	1.831 (1.194–2.807)	*0.006*	1.599 (1.007–2.540)*	*0.047*
Blastocyst morphology (BQS)				
Good	0.445 (0.289–0.685)	*0.001*	0.484 (0.286–0.819)*	*0.007*
Fair	0.577 (0.399–0.835)	*0.004*	0.606 (0.399–0.918)*	*0.018*
Poor	Reference		Reference	
Blastulation day				
Day 5	1.820 (1.130–2.930)	*0.014*	2.017 (1.163–3.498)*	*0.013*
Day 6	Reference		Reference	
Blastocyst re-expansion speed (μm/min)				
≤50 μm/min	Reference		Reference	
*≥*50.1 μm~≤100 μm/min	1.610 (1.067–2.430)	*0.023*	n/a	
≥100.1 μm/min	2.057 (1.331–3.178)	*0.001*	1.996 (1.220–3.268)*	*0.006*
Embryo string				
Yes	1.416 (1.035–1.939)	*0.030*	1.433 (1.013–2.028)*	*0.042*
No	Reference		Reference	

n/a, not applicable.

*Adjusted odds ratios are reported for the variables in the final logistic regression model after backward stepwise selection.

### 3.3 Development of a nomogram for predicting ongoing pregnancy outcomes in SVBT cycles

**Figure 2:**
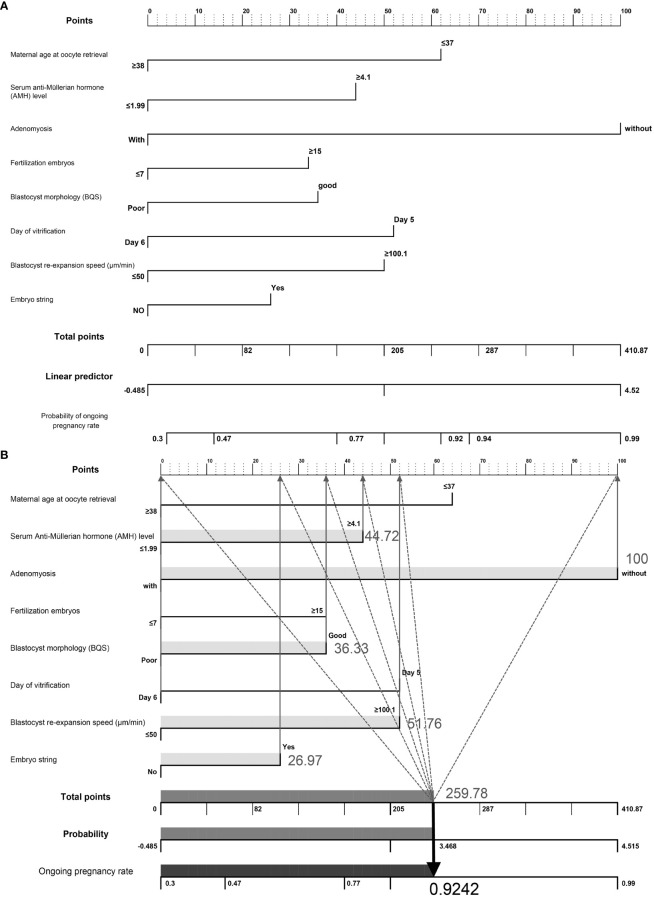
**(A)** Visualization of a nomogram to predict the probability of ongoing pregnancy in patients with infertility undergoing vitrified-warmed single embryo transfer. **(B)** Example of nomogram prediction for ongoing pregnancy rate. A couple with assisted reproductive technology treatment: maternal age at oocyte retrieval, ≥38 years (0 points); anti-Müllerian hormone level, 4.1 (44.72 points); without adenomyosis, (100 points); fertilized embryos, ≥ 7 (0 points); BQS, good (36.33 points); day of vitrification, day 6 (0 points); blastocyst re-expansion speed, 100.1 (51.76 points); presence of embryo string, (26.97 points). The cumulative score of the various predictive indicators was 44.72 + 100 + 36.33 + 51.76 + 26.97 = 259.78, and the corresponding predicted ongoing pregnancy rate was 0.9242 (92.42%).

We developed a nomogram to predict the probability of achieving ongoing pregnancy, as summarized in **Figures 2A, B**, which display the regression coefficients of the eight variables. Each factor is scored and represented by a line below the corresponding score. The length of each line is based on the magnitude of the regression coefficients. The total score represents the sum of all factor scores, and the corresponding probability values are shown. Adenomyosis had the greatest impact on ongoing pregnancy, as indicated by the longest line in the figure. An example of nomogram prediction for the ongoing pregnancy rate is illustrated in **Figure 2B**. For a couple undergoing ART treatment, the maternal age at oocyte retrieval was ≥38 years (0 points), the AMH level was 4.1 (44.72 points), the absence of adenomyosis was at 100 points, fertilization of ≤7 embryos was at 0 points, good blastocyst quality score was 36.33 points, vitrification on day 6 was at 0 points), blastocyst re-expansion speed of 100.1 was at 51.76 points, and the presence of embryo string was at 26.97 points. The cumulative score based on these various prediction indicators was 259.78, resulting in a predicted ongoing pregnancy rate of 0.9242 (92.42%).

### 3.4 Nomogram validation

**Figure 3:**
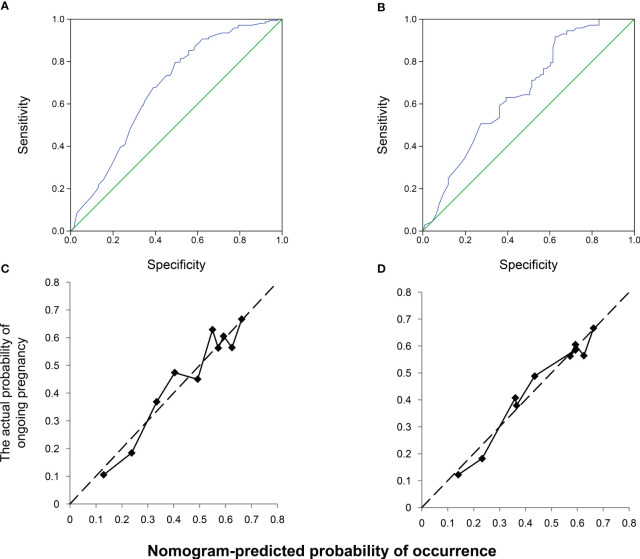
Receiver operating characteristic (ROC) and calibration curves of the nomogram. Discrimination between the training and validation cohorts. **(A)** ROC curve of the training modeling cohort: the area under the curve of the predictive model was 0.675 (95% confidence interval [CI], 0.622–0.729); **(B)** AUROC of the model, 0.656 (95% CI; 0.573–0.739); calibration curves of the nomogram in the **(C)** training and **(D)** validation groups.

The nomogram was validated using the AUROC and a calibration plot to determine the optimal threshold point. ROC curves were used to assess the nomogram prediction accuracy (**Figure 3**). In the training cohort, the AUROC was 0.675 (95% CI, 0.622–0.729), indicating good performance with a 79.85% sensitivity and 50.49% specificity (**Figure 3A**). The validation cohort had similar results, with an AUROC of 0.656 (95% CI, 0.573–0.739), a 91.79% sensitivity, and a 37.36% specificity (**Figure 3B**). The slopes of the calibration plots in the training and validation cohorts demonstrated good agreement between the predicted and ideal lines (**Figures 3C, D**).

## 4 Discussion

### 4.1 Principal findings

In our study, we conducted MLR analysis and identified eight significant variables that were independent predictors of ongoing pregnancy outcomes. These predictors included maternal physiology (maternal age at oocyte retrieval and serum AMH level), uterine factor (adenomyosis), and embryo assessment (number of fertilized embryos, blastocyst morphology, blastulation day, blastocyst re-expansion speed, and presence of embryo string). Using these predictors, we developed a prediction model that combined ongoing pregnancy outcome characteristics, which was validated in both training cohorts. This model demonstrated that ongoing pregnancy outcomes can be predicted by combining the key characteristics of maternal physiology, uterine factors, and major embryonic features. By estimating personalized probabilities and guiding potential treatment options for patients with infertility, our approach can improve the success of *in vitro* treatments. Our findings provide essential data for patient counseling, enabling attending physicians and embryo experts to generalize and provide clinically effective patient counseling.

### 4.2 Clinical interpretations of analytic variables

#### 4.2.1 Maternal age at oocyte retrieval

Maternal age, a widely recognized factor, negatively affects pregnancy outcomes in numerous prior studies (10, 21, 22). In a recent study, maternal aging significantly affected embryonic development from oocyte maturation to blastocyst formation (21). Additionally, a meta-analysis indicated that as the women advance in age at the time of ART, the pregnancy rate decreases by 6% (23). Our findings are consistent with those of previous studies demonstrating that maternal age is crucial for pregnancy outcomes, which, according to Ottosen et al., is unsurprising (24). Therefore, promoting childbirth at a younger age is recommended to enhance the likelihood of successful childbirth and mitigate the adverse effects of age-related decline in fertility. Additionally, future research should prioritize advancements to tackle this challenge and instigate a comprehensive reassessment of prevailing methodologies.

#### 4.2.2 Serum AMH level

According to a systematic review by Broer et al., AMH was proposed as the best predictor of poor response after IVF to at least the same extent as AFC (25). Furthermore, Tremellen et al. reported that serum AMH levels correlate with the ovarian response to gonadotropin stimulation during IVF, making it a widely used ovarian reserve marker (26). Balachandren N’s study also revealed a positive correlation between higher AMH levels (22.1 ρmol/L vs. 10.5 ρmol/L) and cumulative live birth after one cycle of IVF and the transfer of all frozen embryos (27). Consistent with previous studies, our findings indicated that higher serum AMH levels increase the probability of ongoing pregnancy. Conversely, lower levels of AMH, <1.99 ng/mL, negatively affected ongoing pregnancy rates. However, as emphasized in a recent review article by Kotlyar et al., the predictive value of AMH as a qualitative marker for oocyte and pregnancy outcomes remains unclear (28). Therefore, further research is required to investigate the role of AMH as a qualitative marker and its impact on pregnancy outcomes.

#### 4.2.3 Uterine factor

This study identified an association between uterine factors, specifically adenomyosis, and pregnancy outcomes. Recently, Wu et al. developed a validated model for predicting live births in patients with adenomyosis undergoing FET, where a uterine volume > 102.02 cm³ was a significant predictor (29). Similarly, Li et al. reported a uterine volume cut-off of > 98.81 cm³ as a predictor of lowered live birth rate in patients with adenomyosis undergoing FET (30). Another study reported a low success rate and a significantly higher miscarriage rate following IVF in women with adenomyosis than in those without adenomyosis (31). Adenomyosis causes adverse pregnancy outcomes by affecting uterine contractility, endometrial function, and receptivity (32). Previous studies have revealed that an optimal uterine length is associated with increased pregnancy rates, while an excessively large uterus with high volume decreases the clinical pregnancy rate (33, 34). However, our study identified a significant difference in the ongoing pregnancy rate based on the presence of adenomyosis only. In contrast, other factors, such as uterine length and volume, did not significantly correlate. These different and partly conflicting study results may be attributed to varying study designs and population sizes. Further research using more reliable measurement techniques is required to explore the racial and regional correlations of uterine factors in greater detail.

#### 4.2.4 Number of fertilized embryos

This study’s results suggest that an increased number of fertilized embryos is associated with a higher rate of ongoing pregnancies. A greater number of oocytes and fertilized embryos leads to the development of high-quality blastocysts (35, 36). Moreover, large-scale national data (SART data) have demonstrated that up to five blastocysts increased the chances of pregnancy and childbirth. In contrast, subsequent increases were related to a decreasing trend in pregnancy and childbirth rates (37). Additionally, one study proposed that a goal of 10 oocytes is reasonable for maintaining a balance between patient safety and efficiency (38). This study focused only on patients who underwent FET; nonetheless, the results partly concur with those of previous studies. No proportional relationship was observed between the number of retrieved eggs and blastocysts produced; however, more fertilized embryos led to an improved pregnancy rate. Opportunities for conception and childbirth differ significantly across institutions and locations, depending on various ART factors. Therefore, a comprehensive examination of the relationships among the patient, culture, and transplant conditions, and other factors is necessary.

#### 4.2.5 Blastocyst morphology

Over the past few decades, our understanding of embryonic development, conception, and pregnancy has significantly increased. Furthermore, many studies have highlighted the fundamental and crucial role of morphological characteristics as predictors of embryo selection (39–41). Previous studies have suggested that top-quality embryos with high-quality characteristics maintained after warming increase the probability of pregnancy while maintaining their morphological features (42–44). Thus, high-quality embryo transfer is the most critical factor in live birth rates. Our previous study also demonstrated that good-quality embryos exhibit high clinical characteristics, and their importance has been continually emphasized (17–19). However, recent studies have revealed that embryo selection based solely on morphological characteristics is limited in predicting the possibility of pregnancy (45). Therefore, future research should focus on selecting good embryos to provide more objective and quantitative measurements, resulting in greater insights into evaluating their usefulness in improving clinical outcomes.

#### 4.2.6 Blastulation day

With advancements in culture technology, blastocyst transfer is now commonly performed in many IVF centers (46). Regarding development, some embryos reach the blastocyst stage on day 5 of the culture, while others develop into blastocysts on day 6 or 7 (47, 48). Each embryo has characteristics that make its development dynamic. Studies have compared the IVF outcomes of day 5 and day 6 embryo transfer to determine the more efficient embryo (49, 50). However, the optimal approach remains elusive and unclear (48, 51–53). Some studies have suggested that the development time of the blastocyst stage is crucial, whereas others have emphasized the quality of the blastocyst stage over the vitrification time. Our results revealed that day 5 frozen embryos were more efficient than day 6 embryos. However, a binary comparison between freezing time and embryo quality criteria is insufficient. Further research is needed to identify other factors that may influence blastocyst development and analyze their sub-correlations more thoroughly. Therefore, in-depth studies on the relationship between freezing time and quality are required.

#### 4.2.7 Blastocyst re-expansion speed

Recently, studies have focused on identifying key factors that affect the morphological parameters of embryos during the early developmental dynamic processes using practical analyses based on time-lapse imaging. Traditionally, in many studies predicting embryonic characteristics post-warming, re-expansion speed has been highlighted as a crucial parameter, and Ahlstrom et al. identified it as the most important parameter for prioritizing embryo transfer, as embryos with >60% viability within 2–4 h should be transferred first (39). In addition, Shu et al. reported that 78% of embryos displayed re-expansion within 3–4 h of thawing (54). Similarly, studies have uncovered a strong correlation between the maximum and minimum cross-sectional axes measurements and re-expansion outcomes (55–58). Consistent with previous studies, our use of TLS imaging revealed that embryos with faster developmental speeds, as indicated by the quantitative measurement of re-expansion speed, yielded better results.

#### 4.2.8 Cytoplasmic (blastocyst) string

The function of cytoplasmic strings (CS) during early embryonic development remains poorly understood. They generally consist of long, thin projections (0.1–0.3 mm) that connect the inner cell mass and trophectoderm during blastocyst formation (59). Some studies have suggested the importance of CS in human blastocysts; however, current literature indicates that its significance may be limited, and other studies have observed no negative effects (60). In mice, CS promotes cell expansion and migration and is rich in actin (59). Ebner et al. observed a possible association between CS and blastocoel collapse and morphological features in human studies but uncovered no adverse effects on live birth rates or neonatal outcomes (60). Conversely, Eastick et al. proposed a possible relationship between the presence of CS in human blastocysts and the cumulative pregnancy rate (61, 62). Our results revealed that one or more CS were observed in good-quality embryos, with greater early- and mid-stage observations, which disappeared as expansion progressed. The shape and width of the CS vary with the degree of expansion. Our findings are consistent with those of Eastick et al., indicating that CS appear dynamically in the most potentially viable embryos within a short period during the early stages.

### 4.3 Highlight and interpretation of findings

Recently, infertility treatments have increasingly relied on ART and personalized procedures, reflecting the evolution of the medical paradigm toward evidence-based medicine. Emotional understanding between medical professionals and patients is essential to ensure safety, efficiency, and a reduced time to achieving pregnancy. The commonly applied one-size-fits-all IVF treatment may not be suitable for women with infertility who desire to conceive because of the complex interactions between each individual’s endocrine function, embryo status, and uterine factors. Our research proposes a personalized approach that comprehensively considers three factors, maternal physiology, intrauterine conditions, and embryo evaluation, to provide evidence-based and comprehensive information that meets the needs of patients and fosters a trusting relationship. Pregnancy is a continuous process; therefore, continuous collection and investigation of factors affecting pregnancy outcomes is essential. More rigorous personalized tools will assist more patients in establishing healthy families through IVF in the future, thereby increasing the overall success and utilization rates of ART.

### 4.4 Strengths and weaknesses of the study

This study has several strengths, including the comprehensive evaluation of the combined impact of clinical and biological characteristics on the ongoing pregnancy rate in women undergoing SVBT, which has been challenging to identify in previous ART studies. Furthermore, this study highlights the patterns and significance of multiple variables for prediction, confirming a positive association between maternal physiology, uterine factors, embryo assessment, and pregnancy success. This study revealed that these variables are basic and practical factors that can be easily obtained in the laboratory, making them user-friendly combinations of clinical and embryonic morphological characteristics.

The results of studies conducted in the UK and Italy were similar (63, 64). The model developed and validated in our study demonstrated some discriminatory ability; however, it did not demonstrate substantial superiority over other validation models. Numerous factors can influence pregnancy outcomes, and our study may have limitations in capturing all of them, particularly male sperm and genetic influences. In other words, this limitation could be attributed to the unpredictable nature of cycle characteristics and the limitations of retrospective data. Therefore, prospective studies are necessary for reliable validation. Additionally, the nomogram was developed based on data obtained from a single center and internally validated; thus, further external validation at multiple centers is required. Furthermore, innovative approaches and models should be tested using routine measures in clinical practice, and new variables and treatment approaches should be individualized.

## 5 Conclusion

**Figure 4:**
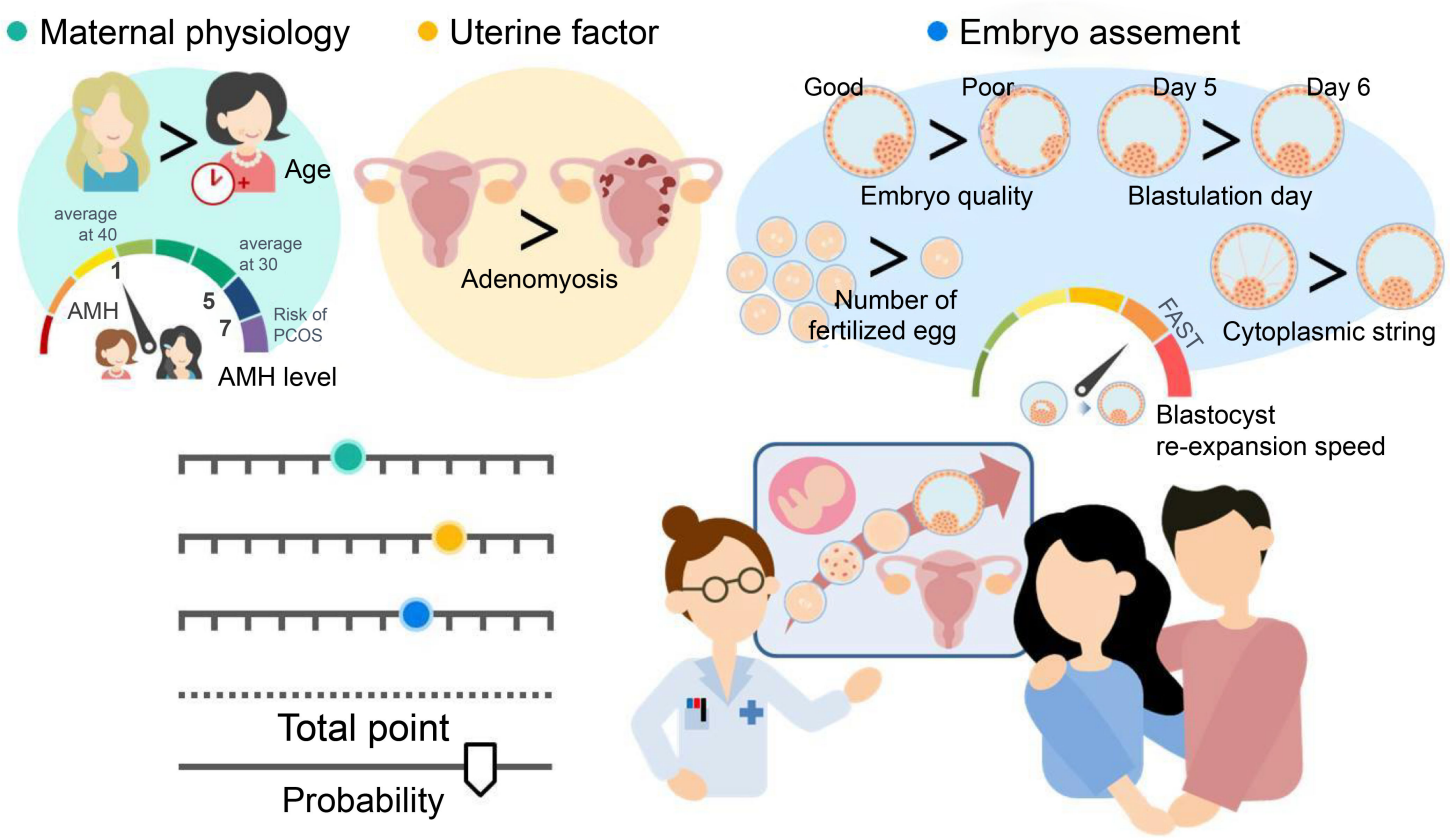
The concept derived from all the data obtained. Our results reveal that the nomogram is a user-friendly tool that combines readily available clinical and biological characteristics, such as maternal physiology (maternal age at oocyte retrieval and serum anti-Müllerian hormone [AMH] level), uterine factors (adenomyosis), and embryo assessment (number of fertilized embryos, blastocyst morphology, blastulation day, blastocyst re-expansion speed, and presence of embryo string). Shared decision-making involves exchanging information between patients and physicians based on objective data to facilitate understanding.

Eight critical predictors were identified from a pool of 28 candidate variables, with clinical perspective being critical in the models. The resulting nomogram is a user-friendly tool that integrates readily available clinical and biological characteristics, such as maternal physiology, uterine factor, and embryo assessment, to predict ongoing pregnancy outcomes in infertile patients undergoing SVBT. Moreover, it offers more informative predictions than continuous pre-cycle pregnancy rates in routine practice. Sharing this model with patients can alleviate uncertainty and confusion regarding fertility treatment options, thereby increasing confidence in making informed decisions. The findings of this study are summarized in **Figure 4**.

## Data availability statement

The original contributions presented in the study are included in the article/**Supplementary Material**. Further inquiries can be directed to the corresponding authors.

## Ethics statement

The studies involving humans were approved by Institutional Review Board (IRB) of the CHA Gangnam Medical Center (GCI 2022-06-008-002). The studies were conducted in accordance with the local legislation and institutional requirements. Written informed consent for participation was not required from the participants or the participants’ legal guardians/next of kin in accordance with the national legislation and institutional requirements. Written informed consent was obtained from the individual(s) for the publication of any potentially identifiable images or data included in this article.

## Author contributions

JKP: Conceptualization, Data curation, Formal Analysis, Investigation, Methodology, Project administration, Validation, Writing – original draft, Writing – review & editing. JEP: Conceptualization, Data curation, Writing – original draft, Writing – review & editing, Validation, Formal Analysis. SB: Writing – review & editing, Investigation, Validation. HJJ: Writing – review & editing, Formal Analysis. JWK: Conceptualization, Data curation, Formal Analysis, Funding acquisition, Project administration, Supervision, Validation, Writing – original draft, Writing – review & editing. WSL: Conceptualization, Methodology, Project administration, Supervision, Writing – original draft, Writing – review & editing.
