# Supplementary material for: Development and validation of a nomogram for predicting ongoing pregnancy in single vitrified-warmed blastocyst embryo transfer cycles
Source: Front Endocrinol (Lausanne). 2023 Nov 23;14:1257764. doi: 10.3389/fendo.2023.1257764 (PMC10702135; doi:10.3389/fendo.2023.1257764)
Supplement: Supplementary file 1 [file DataSheet_1.docx]

Supplementary Material and Methods

**Evaluation of basal hormone levels and ultrasonic measurement**

Whole blood samples were collected before the beginning of COS, on days 2–3 of the menstrual cycle, to determine basal levels of sex hormones such as anti-Müllerian hormone (AMH), follicle-stimulating hormone (FSH), luteinizing hormone (LH), and estradiol (E_2_). An experienced Medical Laboratory Technologist measured the basal hormones using the COBAS 6000 analyzer (Roche) for the same immunoassay. A chemiluminescent immunoassay was used to detect these hormones strictly according to detection instructions. Two experienced sonographers performed ultrasonic measurements using the Voluson TM E10 machine (GE, Boston, MA, USA). All infertile women were routinely examined following a standard method. Furthermore, experienced physicians confirmed all identified cases of adenomyosis. This study included the following parameters: endometrial thickness (EMT), the maximal distance from one endometrial-myometrial interface to the other in the mid-sagittal plane of the uterus; uterus position (anteverted or retroverted); uterine length (mm); uterine anteroposterior diameter (mm); uterine width (mm); uterine volume; EM pathologic findings such as myoma and adenomyosis; and cul-de-sac fluid (CDSF). All patients routinely underwent a transvaginal ultrasound examination the day before the embryo transfer.

**Supplementary methods**

For each variable, a visual examination was conducted using histograms to assess the conformity of the data with a normal distribution. Furthermore, the normality of the data was confirmed using the Kolmogorov–Smirnov test. Following that, quartiles were employed to evaluate the range and central tendency of the dataset. Additionally, the data were partitioned into equalized segments, and the ranges were applied to each respective segment.

# Phase 1: Maternal physiology

This phase included 11 variables: female age (at each oocyte retrieval and embryo transfer); female body mass index (BMI); duration of infertility; number of previous *in vitro* fertilization (IVF) attempts; gravidity; basal FSH, basal E_2_, and serum AMH levels; antral follicle count; endometrial preparation protocols; and endometrial thickness. Participants were divided into maternal age groups: ≤ 37 and ≥ 38 years at oocyte retrieval and ≤ 30, 31–34, 35–37, and ≥ 38 years at frozen embryo transfer. Participants were also divided into three BMI groups: underweight (BMI < 18.5 kg/m^2^), normal weight (18.5 ≤ BMI < 24.99 kg/m^2^), and overweight (BMI ≥ 25 kg/m^2^), with the overweight group as the reference. Participants were divided into three groups according to the duration of infertility: < 2 (reference), 2–3, and ≥ 4 years. Participants were divided into two groups based on the number of previous IVF attempts: ≤ 1 (reference) and ≥ 2. The participants were divided into two groups based on gravidity: yes or no (reference). The participants were categorized into four groups according to basal FSH levels: ≤ 5.9 (reference), 5.91–7.0, 7.01–8.37, and ≥ 8.38. Basal E_2_ levels were classified using quartiles: ≤ 32.4 (reference), 32.5–41.4, 41.5–52.0, and ≥ 52.1. Serum AMH levels were classified into three groups: ≤ 1.99 (reference), 2.0–4.0, and ≥ 4.1. Endometrial preparation protocols were divided into two categories: natural or hormone replacement treatment (reference), and endometrial thickness was classified using quartiles: ≤ 8 (reference), 8.1–9.0, 9.1–11.0, and ≥ 11.1.

# Phase 2: Uterine factor

The following variables were assessed in the uterine cavity: uterine position (anteverted uterus [AV] or retroverted uterus [RV]), uterine length (mm), uterine anteroposterior diameter (mm), width (mm), uterine volume, EM pathology findings, presence of myoma, presence of adenomyosis, and CDSF. Participants were categorized into two groups based on uterine position: AV or RV, based on a previous study (reference). The uterine length had four categories: ≤ 72, 72.1–78.0, 78.1–85.0, and ≥ 85.1 (reference). Uterine anteroposterior diameter (mm) was divided into four categories: ≤ 37.0, 37.1–43.0, 43.1–47.0, and ≥ 47.1 (reference). Width (mm) was classified into four groups: ≤ 44.0, 44.1–48.0, 48.1–53.0, and ≥ 53.1 (reference). Uterine volume was calculated using the formula long diameter anteroposterior width* π/6 (reference) and classified using quartiles: ≤ 56.1, 56.2–73.9, 74.0–96.2, and ≥ 96.3 (reference). EM pathology findings (yes or no), presence of myoma (yes or no), presence of adenomyosis (yes or no), and CDSF results (yes or no) were also assessed.

# Phase 3: Embryo assessment

The following variables were assessed: number of retrieved oocytes (n), number of fertilized embryos (n), fertilization method, number of vitrified blastocysts (n), total vitrification (freeze-all strategy or surplus), blastocyst quality score (BQS), day of vitrification, blastocyst diameter (μm), blastocyst re-expansion speed (μm/min), and presence of embryo string (yes or no). Participants were divided into four groups according to the number of retrieved oocytes (n): ≤ 10 (reference), 11–15, 16–22, and ≥ 23. Four groups were considered according to the number of fertilized embryos (n): ≤ 7 (reference), 8–10, 11–14, and ≥ 15. The fertilization methods were divided into IVF, intracytoplasmic sperm injection (ICSI), and half ICSI (reference). The number of vitrified blastocysts (n) was classified into the following quartiles: ≤ 1 (reference), 2–3, 4–5, and ≥ 6. Total vitrification was divided into two categories: freeze-all strategy and surplus (reference). The BQS was classified as good or poor, and the day of vitrification was categorized as day 5 or 6. The blastocyst diameter (μm) was classified as ≤ 135 (reference), 135.1–144.0, 144.1–153.0, and ≥ 153.1. The blastocyst re-expansion speed (μm/min) was classified as ≤ 50, 50.1–100.0, and ≥ 100.1. The presence of an embryonic string was categorized as yes or no.
